# Use of an objective structured clinical examination (OSCE) to assess intern performance in an advanced pharmacy practice experiences (APPE) Ambulatory Care rotation

**DOI:** 10.1016/j.jsps.2021.10.006

**Published:** 2021-10-25

**Authors:** Ibrahim Sales, Ghada Bawazeer, Mansour Adam Mahmoud, Majidah A. Aljohani, Haya M. Almalag, Abdulaziz Alhossan, Bashayr Alsuwayni

**Affiliations:** a Department of Clinical Pharmacy, College of Pharmacy, King Saud University, P.O. Box 2454, Riyadh 11451, Saudi Arabia; b Department of Clinical and Hospital Pharmacy, College of Pharmacy, Taibah University, Mohamed bin Naif Road, Al-Madinah Al-Munawarah, 42353, Saudi Arabia; c Pharmaceutical Care Division, King Saud Medical City, Al Imam Turki Ibn Abdullah Ibn Muhammad, Ulaishah, 12746, Riyadh; d Corporate of Pharmacy Services, King Saud University Medical City, Riyadh, Saudi Arabia

**Keywords:** Objective structured clinical examination, Ambulatory care, Chronic disease states, Clinical competency, Competency assessment, Experiential Education, Advanced pharmacy practice experience, Student perception

## Abstract

**Purpose:**

Intern assessment during advanced pharmacy practice experiences (APPEs) are generally based upon each individual preceptor’s perceptions without an objective measurement of intern understanding and performance. Therefore, we sought to determine whether a pre- and post-OSCE could be used to confirm that interns achieved the goals and objectives of the Ambulatory Care rotation. The aims of this study were to determine whether a pre-rotation OSCE can help pharmacy interns self-assess their clinical strengths and weaknesses and assess whether their knowledge and skills improved after completing a post-rotation OSCE.

**Methods:**

Pharmacy interns undergoing APPE Ambulatory Care rotations from September 2018 to March 2020 participated in a pre- and post-rotation OSCE to assess their knowledge of various chronic disease states. Interns completed pre- and post-OSCE surveys to assess their perceptions about their knowledge and the OSCE experience.

**Results:**

Pharmacy intern knowledge about diabetes, hypertension, dyslipidemia, and atrial fibrillation significantly improved post-OSCE compared to their pre-OSCE scores (p < 0.001). The mean post-OSCE scores for diabetes (p < 0.001), dyslipidemia (P = 0.046), anticoagulation (P = 0.006), and the overall mean post-OSCE scores (P = 0.005) were significantly higher compared to interns’ pre-OSCE scores. Students believed that the post-OSCE significantly highlighted their strengths and weaknesses in skills and knowledge compared to the pre-OSCE (P = 0.008).

**Conclusion:**

Pre- and post-APPE OSCE assessments are important tools that can provide interns and preceptors with objective evaluations of student performance. OSCEs can either be used as an alternative to perception-based assessments or integrated into existing preceptor evaluations. Furthermore, OSCEs can help preceptors identify areas that require more emphasis in their rotations.

## 1 Introduction

In the last decade as healthcare systems evolved, pharmacy education has expanded rapidly to prepare graduates to engage in a more patient-oriented form of pharmacy practice (Shirwaikar, 2015). With these changes in mind, programs must adapt to prepare students to advance their clinical skills and knowledge. This can be accomplished by incorporating appropriate practice experiences in different settings to allow students to develop, practice, and refine the skills they have learned in the classroom. (Accreditation Council for Pharmacy Education 2016)

The Doctor of Pharmacy (PharmD) curriculum at the King Saud University (KSU) College of Pharmacy is comprised of five years of preparatory and pharmaceutical didactic courses, introductory pharmacy practice experiences (IPPEs), and a final sixth year of advanced practical internships. It was the first international program granted the Accreditation Council for Pharmacy Education (ACPE) International Program Certification in 2013 (Accreditation Council for Pharmacy Education, 2021, Sales et al., 2019). The internship year consists of ten five-week cycles of advanced pharmacy practice experiences (APPEs). As opposed to the limited exposure during IPPEs, APPEs offer the opportunity for interns to become fully immersed in pharmacy practice (Accreditation Council for Pharmacy Education, 2016).

APPEs have been structured to prepare students for clinical practice. They are designed to allow students to develop their professional skills by integrating and applying the knowledge and skills learned throughout the curriculum. Furthermore, APPEs expose interns to direct interactions with patients in collaboration with other healthcare professionals (Accreditation Council for Pharmacy Education, 2016, McLaughlin et al., 2015, Medina et al., 2008). In accordance with the ACPE Standards, pharmacy schools are encouraged to focus more on assessing and evaluating these skills within the lab-based pharmacotherapy courses. (Accreditation Council for Pharmacy Education, 2016) Through these assessment strategies, pharmacy schools and colleges can modify and adapt their curricula to fulfill students’ needs and strive to attain the goals of pharmacy education (Accreditation Council for Pharmacy Education, 2016).

An Objective Structured Clinical Exam (OSCE) is a well-established, standardized tool and has been used for decades to assess pharmacy students’ competency (Gortney et al., 2015, SHIRWAIKAR, 2015). McLaughlin et al. assessed the relationship between admissions, OSCE, and APPE scores. Although a weak association between OSCE scores and APPE performance was confirmed, detecting low OSCE scores early may identify suitable APPEs for prospective interns (McLaughlin et al., 2015). APPE interns are exposed to many patient care encounters as well as interprofessional interactions; therefore, the impact of a pre- and post- OSCE for APPE interns is expected to extend beyond skills development and directly affect patient outcomes (Medina et al., 2008).

APPE rotation evaluations are usually formative and students are given direct and consistent feedback on their performance. Interns’ knowledge deficiencies are typically identified during topic discussions and patient interactions that occur over the course of the rotation. A final evaluation is submitted at the end of the rotation to assess the learning experience, preceptor, site, and intern performance, but this process has been criticized for providing suboptimal and potentially biased assessments (Manning et al., 2016, Martin et al., 2020, Ried and Douglas, 2015, Tofade et al., 2018).

It was hypothesized that providing interns with a pre-rotation OSCE would help to identify knowledge deficiencies early in the rotation; therefore, the rotation experience could be tailored to each interns' particular needs. In addition, a post-rotation OSCEs may help determine whether the intern improved in his/her clinical skills and could be used as a part of the final evaluation. Therefore, we sought to determine whether a pre- and post-OSCE could be used to confirm that interns achieved the goals and objectives of the Ambulatory Care rotation. The aims of this study were to determine whether providing APPE pharmacy interns in Ambulatory Care rotations with a pre-rotation OSCE could help interns self-assess their clinical strengths and weaknesses in the management of chronic diseases, assess whether post-rotation OSCE scores increased compared to pre-rotation scores, and determine whether a post-rotation OSCE improved interns’ perceptions of their chronic disease state knowledge.

## 2 Methods

### 2.1 Study design

This was a cross-sectional study conducted at King Khalid University Hospital (KKUH) from September 2018 to March 2020. A retrospective analysis of OSCE data collected as part of a required assessment during APPE Ambulatory Care rotations was performed for all PharmD and Masters in Clinical Pharmacy interns.

### 2.2 Procedure

#### 2.2.1 Ambulatory Care rotation overview

The Ambulatory Care rotation is one of the mandatory APPE rotations. Interns are assigned to practice sites with clinical pharmacists with expertise in chronic disease state management in the ambulatory or primary care settings such as diabetes clinics, anticoagulation clinics, heart failure clinics, rheumatology clinics, pulmonary clinics, and the outpatient pharmacy. The objective of this 5-week rotation is to allow students to develop and explore their roles in an interdisciplinary health team, actively participate in direct patient care including obtaining patient medical and medication histories, perform physical assessments, evaluate drug therapies, develop pharmacy care plans, and monitor patients' therapeutic outcomes under their preceptor’s supervision (Accreditation Council for Pharmacy Education, 2016, Smith, 2021). Interns are expected to communicate with physician and non-physician providers, provide education to patients and healthcare professionals, and research drug information questions received from healthcare professionals and patients. During Ambulatory Care rotations, interns are also expected to participate in a variety of patient care activities with their pharmacy preceptor and the healthcare team. The interns are exposed to many opportunities to enhance and apply their didactic clinical knowledge of the most common chronic diseases seen in the ambulatory setting. The Ambulatory Care rotation is offered by five preceptors. Each preceptor usually has an average of three interns per rotation cycle. The majority of interns are from the KSU College of Pharmacy PharmD program; however, there may be interns from the KSU Masters in Clinical Pharmacy program as well as interns from other local colleges of pharmacy.

#### 2.2.2 Personnel

The facilitators met in the morning prior to each OSCE and would be briefed on the selected case. Facilitators were primarily the Ambulatory Care preceptors, but occasionally included teaching assistants, residents, and interns who had previously completed the OSCE. There was a bank of four clinical cases designed and reviewed by the Ambulatory Care preceptors. Each facilitator would receive a packet with the selected case, model answers, and assessment forms. The facilitators were also given access to insulin pens, Victoza® pens (the only GLP-1 agonist on the hospital formulary), albuterol inhalers, and diabetes supplies (needles and alcohol swabs).

#### 2.2.3 Development

All OSCE cases included the following disease states: diabetes mellitus, hypertension, dyslipidemia, atrial fibrillation (treated with anticoagulation therapy), asthma, and rheumatoid arthritis. Each case consisted of eight domains which were designed to assess interns’ knowledge about the most common chronic diseases seen in the ambulatory care setting at KKUH (diabetes mellitus, hypertension, dyslipidemia, atrial fibrillation, asthma, and rheumatoid arthritis), medication reconciliation, patient interviewing, insulin dose calculation, identifying drug-related problems (DRPs), designation of an evidence-based care plan to resolve DRPs, and patient education. Patient education was general for all disease states; however, interns were specifically prompted to provide counseling about insulin pen administration, metered-dose inhalers, INR interpretation, and warfarin education. Although some cases also involved other disease states such as hypothyroidism, smoking cessation, deep vein thrombosis, and systemic lupus erythematosus, all evaluation forms were the same and assessment of the interns’ knowledge about the additional disease states was not part of the evaluation rubric.

#### 2.2.4 OSCE administration

Interns were informed prior to the beginning of their rotation cycle that they would participate in an OSCE which was typically conducted on the first day of the rotation. Interns were not required to prepare and were encouraged to take the pre-OSCE without reviewing the various chronic disease state guidelines/materials so that their baseline knowledge could be determined. They were informed that their preceptors would receive their initial results. They were also told that their performance on the post-OSCE would not be used as a determining factor alone on their final evaluations; however, their post-OSCE performance would likely reflect their overall rotation performance. The OSCE would be used as a confirmation of their preceptors’ evaluations. Prior to the OSCE, all interns were provided with instructions for the OSCE. Each OSCE assessment lasted for one hour. Interns were given five minutes to review the patient information sheet that contained the patient’s demographics, vitals, laboratory values, and medications. This was followed by a 20-minute interview/medication reconciliation, ten minutes to write their therapeutic plan, five minutes to discuss their plan with their facilitator, and 20 minutes for the facilitator to ask follow-up questions and request that the student demonstrate patient education techniques such as insulin administration. All interns were required to complete pre- and post-rotation OSCE assessments in addition to pre- and post-OSCE surveys to solicit their perceptions about their OSCE experiences.

#### 2.2.5 Student perceptions

Intern perceptions about the OSCEs and the Ambulatory Care rotation were assessed using a validated survey by Awaisu et al. (Awaisu et al., 2007). The survey included student demographics including their level of training, previous clinical rotations, and the last time they reviewed guidelines or similar materials about the chronic disease states commonly encountered in the Ambulatory Care setting. Interns were also asked to rank their knowledge about the most common diseases before and upon completion of the Ambulatory Care rotation using a 5-point Likert scale ranging from 1 (strongly disagree) to 5 (strongly agree).

### 2.3 Ethical considerations

This study was approved as an expedited study by the KSU College of Medicine Institutional Review Board (No. E-20-5283).

### 2.4 Statistical analysis

Continuous variables were presented as mean (standard deviation) and categorical variables as frequency and percentages. Chi-square test was used to test for associations between pre- and post-OSCE scores and different study domains (disease state knowledge, time spent on reading and student perceptions about OSCE). The pre- and post-OSCE mean total scores in each domain were analyzed using the students *t*-test. The Statistical Package for Social Sciences (SPSS) version 22 (IBM Corp, Armonk, NY, USA) was used in the data analysis.

## 3 Results

The study included 82 interns who completed the pre-OSCE from September 2018 to March 2020. The majority of interns had taken a clinical rotation before starting their Ambulatory Care training, and most were PharmD interns. Due to the COVID-19 schedule modifications in March 2020, post-OSCE data for four interns was incomplete; therefore, the data for 78 interns was included in the analysis (Table 1).

**Table 1:** Demographic Characteristics.

	**Pre-OSCE** **N = 82(%)**	**Post-OSCE** **N = 78(%)**
**Level of Training**		
PharmD	78(95.1)	75(96.2)
Master	4(4.9)	3(3.8)
**Previous clinical rotation**		
Yes	70(85.3%)	N/A
No	12(14.7)	N/A

N/A = Not applicable

Intern knowledge about several diseases was significantly improved post-OSCE (Table 2). The percentage of interns who considered their knowledge about diabetes, dyslipidemia, and atrial fibrillation as good or excellent significantly improved post-OSCE compared to their pre-OSCE scores (p < 0.001). There were no statistically significant differences in knowledge about hypothyroidism between pre- and post-OSCE scores Table 3.

**Table 2:** Comparison of disease knowledge pre- and post-OSCE.

**Survey Item**	**Pre**-**OSCE** **N = 82(%)**			**Post-OSCE** **N = 78(%)**			P value*
	Poor/Fair	Average	Good/Excellent	Poor/Fair	Average	Good/Excellent	
How would you rate your knowledge of diabetes?	29(35.4)	40(48.8)	13(15.9)	11(14.1)	19(24.4)	48(61.5)	<0.001
How would you rate your knowledge of hypertension?	8(9.8)	36(43.9)	38(46.3)	3(3.8)	21(26.9)	54(69.2)	0.011
How would you rate your knowledge of dyslipidemia?	23(28)	34(41.5)	25(30.5)	6(7.7)	24(30.8)	48(61.5)	<0.001
How would you rate your knowledge about atrial fibrillation?	35(42.7)	34(41.5)	34(41.5)	20(26)	24(31.2)	33(42.9)	<0.001
How would you rate your knowledge about rheumatoid arthritis?	52(63.4)	23(28)	7(8.5)	40(51.3)	20(25.6)	18(23.1)	0.041
How would you rate your knowledge about asthma?	43(52.4)	26(31.7)	13(15.9)	21(26.9)	31(39.7)	26(33.4)	0.002
How would you rate your knowledge about COPD?	51(63)	22(27.2)	8(9.9)	31(40.3)	31(40.3)	15(19.4)	0.014
How would you rate your knowledge about hypothyroidism?	34(42)	32(39.5)	15(18.5)	25(32)	30(38.5)	23(29.5)	0.216

* Chi-square; Fishers exact test.

**Table 3:** Preparation for the OSCE.

**Survey Item**	**Pre-OSCE** **N (%)**		**Post-OSCE** **N (%)**		P value*
	≤ 3 h	> 3 h	≤ 3 h	> 3 h	
Diabetes	67(89.3)	8(10.7)	65(90.3)	7(9.7)	0.036
Hypertension	71(95.9)	3(4.1)	69(95.8)	3(4.2)	0.646
Dyslipidemia	70(94.6)	4(5.4)	68(95.8)	3(4.2)	0.523
Atrial fibrillation (anticoagulation)	71(95.9)	3(4.1)	66(94.3)	4(5.7)	0.713
Rheumatoid Arthritis	72(97.3)	2(2.7)	66(94.3)	4(5.7)	0.432
Asthma	72(97.3)	2(2.7)	67(95.7)	3(4.3)	0.675
COPD	72(97.3)	2(2.7)	67(95.7)	3(4.3)	0.675

* Chi-square; Fishers exact test.

The time spent on reading and reviewing guidelines and other required reading materials related to diabetes was significantly increased between pre- and post-OSCE sessions (p = 0.036). However, there were no statistically significant differences for any of the other disease states between pre- and post-OSCE scores. (Table 3).

Interns' mean total scores in each domain were compared between both arms. The total mean post-OSCE score was significantly higher for diabetes compared to the pre-OSCE (p < 0.001). Similarly, the mean post-OSCE score was higher for dyslipidemia (P = 0.046) and anticoagulation (P = 0.006) compared to pre-OSCE mean scores. The overall mean post-OSCE scores were significantly higher compared to the pre-OSCE mean total scores (P = 0.005) (Table 4).

**Table 4:** Intern scores in each OSCE domain.

	**Pre-OSCE (N = 82)** **Mean (SD)**	**Post-OSCE (N = 78)** **Mean (SD)**	P value*
Diabetes (25 points)	11.2(5.6)	14.7(5.8)	<0.001
Medication Reconciliation (26 pts)	14.8(6)	16.3(5.4)	0.115
Hypertension (2pts)	1.2(0.8)	1.4(0.7)	0.240
Dyslipidemia (3 pts)	1.6(1.4)	2.1(1.1)	0.046
Rheumatoid Arthritis (2pts)	0.7(0.8)	1.1(0.8)	0.050
Asthma (12 pts)	7.4(3.1)	8.2(2.7)	0.082
Atrial fibrillation (anticoagulation) (11 pts)	4.5(2.2)	5.7(2.6)	0.006
Total score (81points)	40.7(16.3)	48.1(16.1)	0.005

* Chi-square; Fishers exact test.

The interns provided feedback about the OSCE method and administration. The majority of interns in both arms were satisfied with the OSCE experience. Inters were more satisfied with the time allocated for each section post-OSCE compared to pre-OSCE (P = 0.043) (Table 5). Interns also reported that the OSCE significantly highlighted their strengths and weaknesses in skills and knowledge more in the post-OSCE compared to pre-OSCE (P = 0.008).

**Table 5:** Intern perceptions about the OSCE administration.

**Survey Item**	**Pre-OSCE** **N = 82 (%)**			**Post-OSCE** **N = 78 (%)**			P value*
	Strongly disagree/disagree	Neither agree nor disagree	Strongly agree/agree	Strongly disagree/disagree	Neither agree nor disagree	Strongly agree/agree	
The OSCE was well administered	2(2.7)	5(6.7)	68(90.6)	2(2.8)	3(4.2)	67(93)	0.893
The allocated time for each section was appropriate	3(4)	11(14.7)	61(81.3)	3(4.2)	2(2.8)	67(93.1)	0.043
The OSCE highlighted my strength and weaknesses in skills and knowledge	1(1.3)	7(9.3)	67(89.3)	3(4.2)	0(0)	69(95.8)	0.008
I would recommend OSCE workshops prior to each rotation	3(4)	6(8)	66(80.5)	3(4.2)	5(6.9)	64(88.9)	1.000

* Chi-square; Fishers exact test.

## 4 Discussion

The current study represents, to the best of our knowledge, the first evaluation of pharmacy interns’ pre- and post-Ambulatory Care APPE rotation knowledge and skills using an OSCE. The encouraging results offer an alternative to preceptor evaluations as an assessment of student performance. Overall, interns perceived that knowledge of their strengths and areas requiring improvement were significantly improved.

Interns’ knowledge significantly increased in the majority of disease states taught during the rotation. Most likely, this was in proportion to their exposure to the various disease states. The majority of preceptors work primarily in Diabetes Clinics; therefore, diabetes, hypertension, and dyslipidemia were the most common disease states that the interns were exposed to during their rotation cycles. Most interns also had experience working in the anticoagulation clinic; however, hypothyroidism exposure may have differed among the interns. Each intern was provided with feedback on their OSCE performance from their facilitator and/or preceptor. The OSCE was intended to help preceptors and interns identify weaknesses in knowledge early in the rotation cycle. McLaughlin et al. found that students who performed poorly on OSCE assessments significantly performed substandardly in APPE Ambulatory Care rotations compared to their colleagues (McLaughlin et al., 2015). Furthermore, Ragan et al. concluded that student assessment utilizing standardized patients helped to identify students at risk of underperforming in APPE rotations (Ragan et al., 2013). Likewise, it has been suggested that OSCE results can be used by college administrators and preceptors to focus on areas that students do not meet expectations (Curtis et al., 2019, McLaughlin et al., 2015). The pre-OSCE made preceptors aware of each individual intern’s deficiencies; therefore, they could tailor discussions and learning experiences accordingly for interns with lower pre-OSCE scores.

Interns were not required, nor was it preferred for them to read the guidelines for the various disease states prior to the pre-OSCE; however, they were asked to be familiar with the guidelines during their rotations and were responsible for preparing for patient encounters, disease state discussions, and presentations. Since the majority of interns worked with patients with diabetes, the time spent reading the diabetes guidelines was expected to increase significantly during the rotation. The other disease states may have increased, but not to the point that was considered significant to the investigators based upon the average reading hours of physicians and considering the level of the interns (Doximity, 2014).

Overall, mean scores on the OSCE increased significantly from the pre- to post-OSCE sessions. Martin et al. conducted a pre- and post-IPPE OSCE to assess the competency of pharmacy students in their third year of pharmacy school (Martin et al., 2020). Mean post-IPPE scores also improved significantly compared to their initial scores which, along with the results of this study, confirms the advantages of utilizing OSCEs in both IPPE and APPE intern assessments. In our study, intern scores significantly improved in the majority of disease states with diabetes representing the greatest improvement. Scores improved, but not significantly for medication reconciliation, hypertension, and asthma. ntern mean scores increased from 50% on the pre-OSCE to approximately 60% on the post-OSCE (p = 0.005). As for diabetes, this may be attributed to the fact that some interns had disease-specific Ambulatory Care rotations. Some interns had diabetes-focused rotations and only a minority weren’t exposed to diabetes patients at all. As for medication reconciliation, the rubric used in the OSCE may have differed from the approach used in the clinics. This is in addition to the fact that the interns were not provided with a specific form to use during the OSCE. Martin et al. reported a significant increase in IPPE student scores for medication reconciliation; however, at KSU, medication reconciliation is a skill that students repeatedly encountered during their pharmacy practice laboratories, and the grading rubric used for the OSCE was the same rubric used during their laboratory sessions (Martin et al., 2020). It is possible that IPPE students may show a greater improvement in medication reconciliation since it is a skill under development. APPE interns may have already mastered this skill especially those interns who completed other clinical rotations prior to Ambulatory Care. Furthermore, interns rated their pre-OSCE knowledge about hypertension as high. Nearly 50% of students considered their knowledge of hypertension to be good/excellent for the pre-OSCE which was higher than any other disease state; therefore, it makes sense that there wasn’t a significant improvement in their post-OSCE scores. Although interns' knowledge significantly increased about asthma from the pre- to post-OSCE, their mean OSCE scores did not increase significantly. Intern attendance in the respiratory clinics may have varied among rotation cycles and preceptors.

Interns’ perceptions regarding the OSCE administration and outcomes were positive. This confirms that OSCEs are beneficial learning experiences in both IPPE and APPE interns (Curtis et al., 2019, Martin et al., 2020). Although intern satisfaction only increased for their post-OSCE evaluation of the time allocated, they believed that the OSCE was well-organized even during the pre-OSCE sessions. Intern satisfaction regarding the time allocation may have increased due to their familiarity with both the OSCE procedure and the disease state topics. Shirwaikar discussed the advantanges and disadvantages of OSCEs in pharmacy education and concluded with some guidelines to facilitate implementation. She mentions that organizational issues such as coordination and proper administration are necessary to establish the validity of the examination (Shirwaikar, 2015). Furthermore, the interns believed that OSCE sessions should be used in all APPE rotations. The actualization of this recommendation is feasible since APPE rotations have been likened to OSCEs stations and can vary in their levels of complexity (Peeters and Cox, 2011). In addition, OSCEs allow students and interns the opportunity to be exposed to patient encounters in a safe and stimulating learning environment (Azer et al., 2013).

There are some limitations of this study that should be mentioned. Some ambulatory care rotations were more comprehensive than disease-focused rotations (i.e. diabetes). In disease-focused rotations, intern exposure to other disease states may have been limited. Second, the OSCE sessions were not recorded for quality assurance. Third, although the primary facilitators had previous experience with facilitating OSCE sessions in the college of pharmacy and every new facilitator was met with individually and debriefed prior to participating in the OSCE, there was no organized facilitator training offered. Fourth, the location of the OSCE sessions were scheduled in different meeting rooms throughout the hospital. This may have led to varying degrees of adequacy in seating arrangements during the OSCE. Fifth, the facilitators were responsible for playing the role of a patient and grading the interns simultaneously. This may have led to a lack of full engagement for either role during the session. Sixth, although intern scores were emailed to each preceptor, this did not guarantee that each preceptor used the information to tailor the rotation toward each individual intern's performance. Finally, the majority of interns were from KSU College of Pharmacy; therefore, the results may not be generalized for other universities and colleges.

Future recommendations include scheduling all Ambulatory Care OSCE sessions in the KSU Medical College Simulation Center which will assist in assuring adequate spacing arrangements. Standardized patients/healthcare professionals can also be requested through the center. This will allow the facilitators to focus more on interns' performance and will allow additional feedback to be provided to the intern by the standardized patients/healthcare professionals. Second, the incorporation of other healthcare professionals, such as interns and clinicians, will also facilitate the incorporation of interprofessional interactions within the OSCE. Interprofessional Collaborative Competency Attainment Survey scores were found to be significantly higher after completing capstone OSCE cases (Doloresco et al., 2019). Third, all facilitators should complete mandatory training prior to facilitating OSCE sessions. This will ensure consistency in grading across facilitators. Fourth, a debriefing session should be held immediately after the pre- and post-OSCE sessions to discuss the interns' performance and answer their questions. Fifth, intern performance should be incorporated into their midterm evaluations and final grades. Sixth, disease state topic discussions should be conducted jointly among the Ambulatory Care interns for each cycle to minimize variations in interns' knowledge and exposure to the common chronic diseases. Finally, there should be a system for remediation established for poor performing students.

## 5 Conclusion

This study provides further credence for the use of OSCEs in the assessment of IPPE and APPE interns. OSCE assessments have the potential to be used alternatively to perception-based assessments or integrated into pre-existing preceptor evaluations. Future research should focus upon the use of OSCE assessments in other APPE rotations and the incorporation of both standardized patients and interprofessional scenarios.

## Declaration of Competing Interest

The authors declare that they have no known competing financial interests or personal relationships that could have appeared to influence the work reported in this paper.
